# Heel impact forces during barefoot versus minimally shod walking among Tarahumara subsistence farmers and urban Americans

**DOI:** 10.1098/rsos.180044

**Published:** 2018-03-14

**Authors:** Ian J. Wallace, Elizabeth Koch, Nicholas B. Holowka, Daniel E. Lieberman

**Affiliations:** Department of Human Evolutionary Biology, Peabody Museum, Harvard University, Cambridge, MA 02138, USA

**Keywords:** ground reaction force, heel strike, *huaraches*, impact peak, minimal shoes, *Rarámuri*

## Abstract

Despite substantial recent interest in walking barefoot and in minimal footwear, little is known about potential differences in walking biomechanics when unshod versus minimally shod. To test the hypothesis that heel impact forces are similar during barefoot and minimally shod walking, we analysed ground reaction forces recorded in both conditions with a pedography platform among indigenous subsistence farmers, the Tarahumara of Mexico, who habitually wear minimal sandals, as well as among urban Americans wearing commercially available minimal sandals. Among both the Tarahumara (*n *= 35) and Americans (*n *= 30), impact peaks generated in sandals had significantly (*p *< 0.05) higher force magnitudes, slower loading rates and larger vertical impulses than during barefoot walking. These kinetic differences were partly due to individuals' significantly greater effective mass when walking in sandals. Our results indicate that, in general, people tread more lightly when walking barefoot than in minimal footwear. Further research is needed to test if the variations in impact peaks generated by walking barefoot or in minimal shoes have consequences for musculoskeletal health.

## 1. Introduction

For most of the last six million years, species in the human lineage walked and ran barefoot [1]. Minimal forms of footwear such as sandals and moccasins were probably invented only during the last 50 000 years [2,3], and shoes with sophisticated features such as thick, cushioned heels, toe springs and arch supports became common within just the last half-century [4]. Modern, supportive shoes are popular because they enhance comfort and provide protection, but some of their effects on foot structure and biomechanics may be mismatched with the functional environment for which the human foot evolved [1]. According to this mismatch hypothesis, certain aspects of some shoe designs potentially contribute to the development of weak feet and several lower extremity musculoskeletal disorders including plantar fasciitis, stress fractures and osteoarthritis [5,6]. Although these perceived links between modern shoes and pathology risk are poorly tested, there has been considerable public and scientific interest in the potential health benefits of walking and running barefoot or, more commonly, in minimal footwear [7,8].

Despite widespread interest in purported health benefits of bare feet and minimal footwear, there has been little research on the extent to which locomotor biomechanics differ when unshod versus minimally shod. Although some commercially available types of minimal footwear are marketed (oxymoronically) as ‘barefoot shoes', any type of footwear, no matter how minimal, that covers the sole of the foot will inevitably limit plantar sensory feedback, thus potentially affecting how the foot functions during impact and while in contact with the ground. It is therefore reasonable to expect that walking and running differ to some extent in barefoot and minimally shod conditions [5,9].

Although walking is by far the most common human gait, nearly all previous research on the effects of bare feet versus minimal footwear has focused on running, especially at the moment of impact [5,10,11]. People who habitually wear highly cushioned running shoes most often land with a heel strike [12,13]. Heel strikes generate a rapid peak in the vertical ground reaction force from the nearly instantaneous exchange of momentum between the ground and the effective mass of the lower limb that decelerates completely at impact [13–15]. By contrast, habitually barefoot runners have more variable strike patterns [16] but often land with forefoot or midfoot strikes [13,17,18], which usually do not cause measurable impact peaks due to greater ankle compliance and lower effective mass [10,13,19]. However, it is less clear how minimal shoe use affects running. Two traditional-living indigenous populations who habitually wear minimal sandals, the Tarahumara of Mexico and the Hadza of Tanzania, most commonly midfoot strike [20,21], but an analysis of Americans running in minimal footwear found that nearly 50% used heel strikes [18].

Notwithstanding the focus of prior research on running, the effects of minimal footwear versus bare feet during walking are considerably more relevant for most people. Whereas only approximately 7% of the US population runs recreationally on a regular basis [22], the average American walks approximately 5000 steps per day [23]. Further, almost all walking regardless of footwear involves heel strikes that generate impact peaks [24,25]. To what extent impact peaks, especially during walking, contribute to certain musculoskeletal disorders is highly debated [24,26–28], but two prior studies based on small sample sizes suggested that people generate impact peaks with significantly higher magnitudes when walking barefoot than in cushioned, supportive shoes [29,30]. However, little is currently known about how impact peaks are produced by people walking in minimal footwear. The most potentially relevant data are from Willems *et al*. [31], who used accelerometers mounted on the heel to record foot deceleration following impact among rural South Indians who regularly walk barefoot or in minimal sandals made of buffalo skin. Although ground reaction forces were not measured, this study found little difference in foot deceleration following impact during walking barefoot compared to in sandals, possibly indicating similar impact peaks in the two conditions. The relevance of this study, however, which seems to contradict the findings of the two earlier studies comparing impact peaks while barefoot and in supportive shoes [29,30], is limited by the lack of ground reaction force data, highlighting the need for additional research.

**Figure 1.**
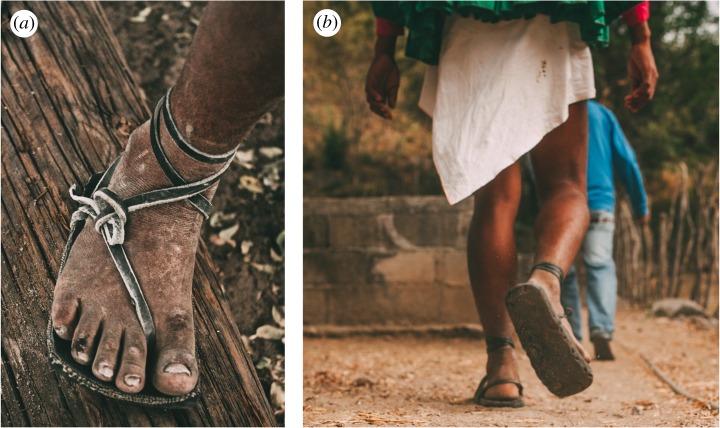
Traditional Tarahumara sandals (*huaraches*). (*a*) Tarahumara man wearing sandals with soles made from car tyres and a leather thong that goes between the first and second toes and wraps around the ankle. (*b*) Tarahumara man walking in traditional sandals. Photos copyright © 2018 by David Ramos and used here with permission. All rights reserved.

Here, to gain insight into how humans generate impact peaks when walking barefoot versus in minimal footwear, we analyse ground reaction forces and limb kinematic data collected from two distinct populations. First, we studied an indigenous population that habitually wears minimal sandals, Tarahumara Native Americans from the Sierra Madre Occidental of Mexico. The Tarahumara (who call themselves the *Rarámuri*) are subsistence farmers who grow and eat mostly maize and beans. Most Tarahumara walk long distances on a regular basis wearing traditional sandals (*huaraches*) that consist only of a piece of stiff material fastened to the bottom of the foot by a leather strap (figure 1). According to early ethnographic accounts, the Tarahumara often used to travel barefoot [32,33], but today barefoot walking is uncommon [20]. Prior to roughly a half-century ago, the Tarahumara fabricated the soles of their sandals from thin strips of rawhide or leather [33]. Today, however, their sandals' soles are mostly made from the tread surfaces of car tyres, a design innovation that has occurred independently among many populations around the world that use minimal footwear [34,35]. In other respects, contemporary Tarahumara sandals differ little from those documented by early ethnographers or those recovered from archaeological sites [33].

To assess if the findings from the Tarahumara are consistent with those obtained from a population that is not habitually minimally shod, we also measured walking kinetics and kinematics in a group of urban Americans while barefoot and in a commercially available brand of minimal sandals with soles made of thin strips of ethylene vinyl acetate (EVA), a material widely used to cushion modern athletic shoe soles [24]. All of these individuals normally wear mostly cushioned, supportive shoes and only rarely walk barefoot or in minimal footwear.

Together, data from the Tarahumara and Americans were used to test two hypotheses independently in two discrete populations. First, in both groups, we tested the hypothesis that aspects of impact peaks (magnitude, loading rate and vertical impulse) generated at the moment of heel strike are similar during barefoot and minimally shod walking. Second, we tested the hypothesis that factors known to influence impact peaks (effective mass, impact velocity and lower limb joint flexion) are also comparable between footwear conditions within each population.

## 2. Material and methods

### 2.1. Participants

A group of 35 Tarahumara men (average (s.d.) body weight (kilogram): 64 (10), height (metre): 1.60 (0.06)) between 41 and 75 years were recruited from the regions around the Sinforosa and Urique canyons in the southwestern portion of the Mexican state of Chihuahua. We limited our recruitment to individuals aged 40 years and older in order to exclude people who do not habitually wear traditional minimal sandals, because many younger Tarahumara today often wear cushioned, supportive shoes [20]. Participants were recruited by word of mouth with the help of local residents and were transported to clinics in the towns of Guachochi and Cerocahui where the experiments were conducted. No individuals who arrived in footwear other than traditional sandals were measured. Individuals who reported or manifested any evidence of current lower extremity injuries or gait abnormalities were also excluded. Although the research questions being addressed apply as much to women as men, we were not able to measure women because of their reluctance to come to the clinic. The American group consisted of 30 men (body weight: 79 (10), height: 1.78 (0.07)) between 40 and 77 years. Data from these participants were collected in the Skeletal Biology and Biomechanics Laboratory in the Department of Human Evolutionary Biology at Harvard University. All participants gave their informed consent in *Rarámuri* (the native language of the Tarahumara), Spanish or English.

### 2.2. Experimental design and measurements

Vertical ground reaction forces were recorded as participants walked at self-selected speeds across a low-profile (1.5 cm) pedography platform (emed-q100, Novel GmbH, Munich, Germany) at a sampling frequency of 100 Hz. Prior to data collection, participants practised walking across the platform until they felt able to consistently strike the force sensor area (48 cm × 32 cm) with their right foot while maintaining a natural steady-state gait (i.e. without shifting their velocity by abruptly shortening or lengthening their steps). No more than 10 practice attempts were required per participant. To prevent under- or over-striding, participants were asked to look forward when they walked, rather than at their feet, which discouraged targeting foot placement on the platform. Participants took at least three steps before and after platform contact to ensure that data were collected from steady-state gait [36,37]. For shod trials, Tarahumara participants wore the traditional sandals that they use on a daily basis, whereas American participants were supplied on the day of the experiment with a commercially available minimal sandal designed to fit similarly to Tarahumara sandals (Mono, LUNA Sandals, Seattle, WA, USA) but made of EVA rather than car tyres. For each participant, data were collected from five barefoot and five shod trials, interspersed with rest ad libitum. Trials were discarded when participants targeted the platform or failed to make contact completely within the force sensor area.

To record kinematics, two video cameras (Hero 4, GoPro Inc., San Mateo, CA, USA) with 7.5 mm 3MP M12 lenses (Back-Bone Inc., Kanata, ON, Canada) were used. One camera that recorded at 120 Hz was positioned at approximately 70 cm height and 2 m lateral to the platform. The second video camera recording at 240 Hz was positioned at approximately 10 cm height and 50 cm medial to the pedography platform. Camera and platform recordings were synchronized using a light on the platform that illuminates at the instant of foot contact. Data from the lateral camera were used to calculate walking speed based on the horizontal translation of a reflective tape marker placed on the right greater trochanter of each participant. In order to measure knee flexion and ankle dorsiflexion angles in the sagittal plane at the moment of heel strike from the lateral camera recordings, lower extremity kinematic data were collected from a subset of participants (*n *= 24 Tarahumara; *n *= 21 Americans). These individuals wore short trousers that exposed their knees with reflective tape markers affixed to the following locations on the right sides of their bodies: greater trochanter, centre of the knee (between the lateral femoral epicondyle and lateral tibial plateau), lateral malleolus and the lateral surface of the fifth metatarsal head (electronic supplementary material, S1). Data from the medial camera were used to measure impact velocity by tracking the position of an additional marker placed on the medial malleolus. Following Lieberman *et al*. [13], impact velocity was calculated as the change in the position of the medial malleolus divided by the change in time for the four video frames immediately prior to heel strike. All camera data were analysed using ImageJ software (v. 1.50i, NIH, Bethesda, MD, USA). Trials were discarded when body markers were obscured in the camera recordings.

For each heel strike, we calculated the impact peak magnitude, loading rate and vertical impulse using Igor Pro software (v. 4.07, WaveMetrics, Lake Oswego, OR, USA). Values were normalized to body weight to facilitate comparisons across individuals [38]. Vertical impulse was calculated as the integral of the impact peak over the time duration of impact (Δ*t*). For trials where impact velocity (Δ*v*) was measured, we also calculated effective mass (*M*_eff_) using the following equation from Lieberman *et al*. [13]:

$$M_{\mathrm{eff}}=\frac{\int_{t_{i}}^{t_{f}}F_{z}\;\text{d}t}{\Delta v+g\Delta t},$$

where *t_i_* and *t_f_* are the beginning and end times of the impact phase, *F_z_* is the vertical ground reaction force and *g* is the acceleration due to gravity.

### 2.3. Footwear stiffness

Although the first hypothesis we test predicts that impact forces are similar during barefoot and minimally shod walking, if this hypothesis is falsified and we find that impact peaks are significantly affected by footwear, then such effects might be expected to vary between the Tarahumara and Americans because the soles of their sandals are made of different materials. Specifically, holding all other variables constant, the effective mass of the lower extremity that exchanges momentum with the ground during impact should decelerate to zero over a longer period of time in whichever type of sandal has a less stiff sole, thus generating a larger vertical impulse [15]. To measure the difference between the stiffness of sandals worn by Tarahumara participants and the commercial sandals worn by the American participants, three pairs of each sandal type were loaded in compression using an Instron 4201 material testing machine (Instron, Norwood, MA, USA) fitted with a 500 N force cell. The Tarahumara sandals were purchased from three participants in the study. Five sections from each sandal were tested by applying a compressive force with a stainless steel cylindrical punch (12 mm diameter) fixed to the crosshead. Maximum displacement was set at 10% of the section thickness. Loading velocity was 3% of the maximum displacement per second. Data were recorded at 100 Hz. Stiffness was calculated from the linear portion of the force–displacement curve.

### 2.4. Statistical analyses

To assess sources of variation in impact peak variables, as well as effective mass, impact velocity and joint angles, we used general linear mixed models (GLMMs) that included footwear condition, population and the footwear condition × population interaction as fixed effects, and participant identity as a random effect. Walking speed was included as a covariate due to its well-documented effect on impact forces [39]. On average, the Tarahumara tended to walk moderately slower than the Americans (average (s.d.) walking speed (m s^−1^): 0.95 (0.17) and 0.96 (0.17) for the Tarahumara based on 83 barefoot and 88 shod steps, respectively, and 1.06 (0.14) and 1.06 (0.15) for the Americans based on 50 barefoot and 56 shod steps, respectively). Tukey HSD *post hoc* tests were used to compare the least-squares means generated for each of the four footwear condition × population states. Mixed model nested analysis of variance was used to compare the stiffness of the Tarahumara and American sandals, with sandal section as a random factor nested within sandal number. Stiffness values were log-transformed prior to analysis to improve data normality and the homogeneity of variances. All statistical analyses were conducted using JMP Pro software (v. 11.0, SAS Institute, Cary, NC, USA), with statistical significance set at *p *< 0.05.

## 3. Results

### 3.1. Footwear stiffness

**Figure 2.**
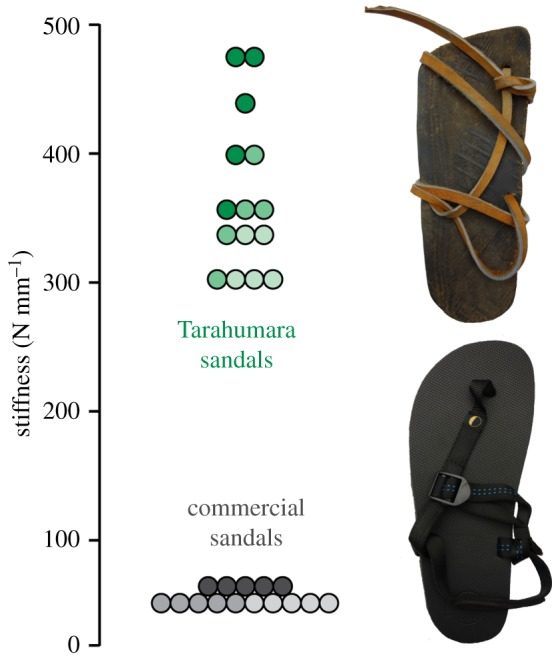
Differences in sole stiffness between traditional Tarahumara sandals made of car tyres and the commercial sandals made of EVA worn by the Americans. Green and grey circles are stiffness values for Tarahumara and commercial sandals, respectively (*n *= 3/sandal type). Circles with the same shade of colour represent different specimens from each sandal tested (*n *= 5/sandal).

Traditional minimal sandals worn by the Tarahumara participants were at least 3.4 times stiffer than the stiffest commercial minimal sandals worn by the American participants (*p *< 0.0001). In compression tests (figure 2), stiffness values obtained from Tarahumara sandals ranged between 300 and 480 N mm^−1^, whereas the average stiffness of commercial sandals was only 55 N mm^−1^.

### 3.2. Heel strike kinetics and kinematics

**Figure 3.**
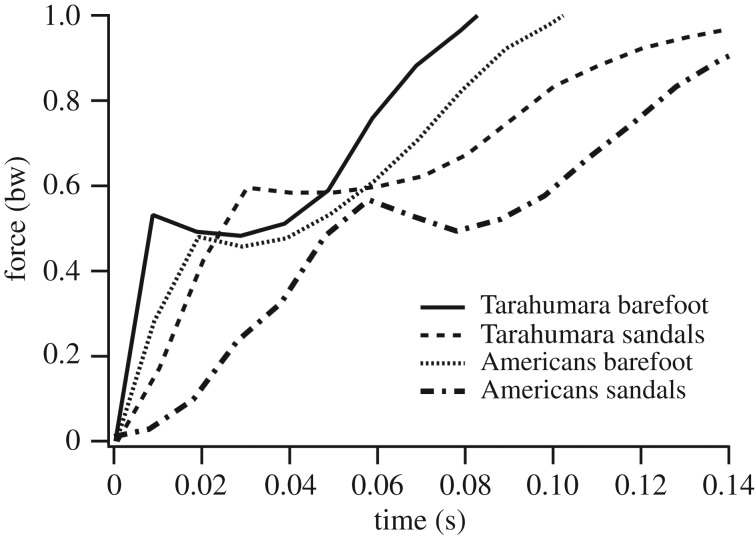
Ground reaction forces produced at the beginning of stance phase by Tarahumara and Americans walking barefoot and in minimal sandals. Impact peak values approximate the least-squares means reported in table 1.

Impact peaks generated by Tarahumara walking in traditional minimal sandals were significantly different than those generated from barefoot walking (tables 1 and 2; figure 3). Whereas Tarahumara barefoot walking produced impact peaks characterized by rapid rates of loading and small vertical impulses, walking in traditional sandals engendered rates and impulses of impact loading that were 41% slower (*p *< 0.0001) and 48% larger (*p *< 0.0001), respectively, after statistically controlling for variation in walking speed. Moreover, the magnitude of impact peaks was 11% higher (*p *< 0.0001) when Tarahumara walked in sandals compared to barefoot. Underlying these effects of footwear on impact peaks was a significant difference in the exchange of momentum between the body and ground, with walking in sandals involving a 32% greater (*p *< 0.0001) portion of the body (effective mass) coming to a stop during the period of impact than in barefoot walking. In terms of joint kinematics, Tarahumara landed with 2% more (*p *= 0.003) dorsiflexed ankles when wearing sandals versus barefoot, but knee angle was not significantly affected by footwear.

**Table 1.** Differences in kinetic and kinematic variables between footwear conditions and populations. Values in cells are least-squares means ± s.e. and numbers of steps analysed. Least-squares means and standard errors were generated by GLMMs that included footwear condition, population, the footwear condition × population interaction and walking speed as fixed effects, and participant identity as a random effect. Impact peak variables and effective mass are in units of body weight (bw). Values not connected by the same letter are significantly different based on Tukey HSD *post hoc* comparisons. Symbols indicate significant effects of footwear condition (†), population (*), the footwear condition × population interaction (‡) and walking speed (§).

	Tarahumara barefoot	Tarahumara in sandals	Americans barefoot	Americans in sandals
impact peak magnitude (bw)^†,§^	0.53 ± 0.02^a^^,^^b^	0.59 ± 0.02^c^	0.48 ± 0.02^b^	0.57 ± 0.02^a^^,^^c^
	83	88	50	56
impact loading rate (bw s^−1^)^†,*,§^	36.2 ± 2.0^a^	21.2 ± 2.0	30.2 ± 2.4^a^	9.8 ± 2.3
	83	88	50	56
vertical impulse (bw * ms)^†,*,‡^	9.5 ± 1.0^a^	14.1 ± 1.0^b^	12.1 ± 1.2^a^^,^^b^	21.4 ± 1.1
	83	88	50	56
impact velocity (m s^−1^)^†,§^	0.40 ± 0.02^a^^,^^b^	0.38 ± 0.02^a^^,^^b^	0.41 ± 0.02^a^	0.36 ± 0.02^b^
	59	56	36	39
effective mass (%bw)^†^	13.8 ± 1.1^a^	18.2 ± 1.1^b^^,^^c^	14.5 ± 1.2^a^^,^^b^	19.4 ± 1.2^c^
	59	56	36	39
knee angle (deg.)	169 ± 1^a^	169 ± 1^a^	172 ± 1^a^	171 ± 1^a^
	68	72	36	40
ankle angle (deg.)^†,*^	128 ± 1	126 ± 1	120 ± 1	117 ± 1
	68	72	36	40

**Table 2.** Effect size of differences in kinetic and kinematic variables between footwear conditions and populations reported in table 1. Values in cells are least-squares mean differences ± s.e. of differences, 95% confidence intervals for the least-squares mean differences in parentheses and *p*-values based on Tukey HSD *post hoc* comparisons.

	Tarahumara barefoot versus in sandals	Americans barefoot versus in sandals	Barefoot: Tarahumara versus Americans	Sandals: Tarahumara versus Americans
impact peak magnitude (bw)	−0.06 ± 0.01	−0.09 ± 0.02	0.05 ± 0.03	0.02 ± 0.03
	(−0.10, −0.03)	(−0.13, −0.04)	(−0.02, 0.11)	(−0.04, 0.09)
	<0.0001	<0.0001	0.28	0.83
impact loading rate (bw s^−1^)	15.0 ± 1.9	20.4 ± 2.4	6.0 ± 3.2	11.4 ± 3.1
	(10.0, 20.0)	(14.1, 26.7)	(−2.2, 14.2)	(3.4, 19.4)
	<0.0001	<0.0001	0.23	0.002
vertical impulse (bw*ms)	−4.7 ± 0.9	−9.3 ± 1.2	−2.7 ± 1.5	−7.3 ± 1.5
	(−7.1, −2.2)	(−12.4, −6.2)	(−6.6, 1.3)	(−11.1, −3.4)
	<0.0001	<0.0001	0.31	<0.0001
impact velocity (m s^−1^)	0.03 ± 0.01	0.05 ± 0.02	0.00 ± 0.03	0.02 ± 0.03
	(0.00, 0.06)	(0.01, 0.09)	(−0.07, 0.06)	(−0.10, 0.04)
	0.12	0.015	0.99	0.94
effective mass (%bw)	−4.4 ± 0.9	−5.0 ± 1.1	−0.6 ± 1.7	−1.2 ± 1.7
	(−6.8, −2.1)	(−7.9, −2.0)	(−5.0, 3.7)	(−5.5, 3.1)
	<0.0001	0.0001	0.98	0.89
knee angle (deg.)	0 ± 1	1 ± 1	−3 ± 1	−2 ± 1
	(−2, 1)	(0, 3)	(−7, 0)	(−5, 2)
	0.86	0.23	0.09	0.68
ankle angle (deg.)	2 ± 1	2 ± 1	8 ± 1	9 ± 1
	(0, 3)	(1, 4)	(4, 12)	(5, 12)
	0.003	0.002	<0.0001	<0.0001

As with the Tarahumara, Americans walking in commercial minimal sandals produced impact peaks that were significantly dissimilar to those produced when barefoot (tables 1 and 2; figure 3). Relative to barefoot, walking in commercial sandals generated rates and impulses of impact loading that were 68% slower (*p *< 0.0001) and 77% larger (*p *< 0.0001), respectively, after statistically accounting for variation in walking speed. Additionally, impact peak magnitudes were 19% higher (*p *< 0.0001) in sandals relative to barefoot. These effects of footwear on impact peaks were caused by a 34% increase (*p *= 0.0001) in effective mass and a 12% decrease in impact velocity (*p *= 0.015) compared to barefoot walking. Moreover, like the Tarahumara, Americans made heel contact with 2% more dorsiflexed ankles when wearing sandals than barefoot (*p *= 0.002), yet knee angle was not significantly altered by footwear.

Although the overall effects of minimal footwear were very similar among the Tarahumara and Americans, significant differences were evident between impact peaks produced by Tarahumara walking in traditional sandals and those generated by the Americans in commercial sandals (tables 1 and 2; figure 3). Compared with the forces generated by Tarahumara heel strikes, American sandal walking elicited impact peaks with rates and impulses of loading that were 54% slower (*p *= 0.002) and 52% larger (*p *< 0.0001), respectively, after statistically controlling for variation in walking speed. Average impact peaks associated with barefoot walking also differed to some extent between populations, but not significantly so (tables 1 and 2; figure 3). In both sandal and barefoot walking, impact peak magnitudes were not significantly distinct between the Tarahumara and Americans, nor were effective mass, impact velocity or knee kinematics. At the moment of heel strike, however, with or without sandals, ankle plantarflexion was 7–8% greater (*p *< 0.0001) among the Tarahumara.

## 4. Discussion

To gain insight into the biomechanics of human walking when barefoot versus in minimal footwear, we collected data on ground reaction forces and limb kinematics from an indigenous population of subsistence farmers from Mexico, the Tarahumara, that habitually wears minimal sandals, as well as from a group of urban Americans wearing a commercially available brand of minimal sandals. Analyses of ground reaction force data focused on multiple aspects of impact peaks (magnitude, loading rate and vertical impulse) generated at the moment of heel strike, which has been hypothesized to play a role in the etiology of certain lower extremity musculoskeletal disorders [5,6]. Ultimately, kinetic and kinematic data gathered from the two populations walking barefoot and in traditional and commercial minimal sandals, respectively, failed to support either the hypothesis that impact peaks do not significantly differ between barefoot and minimally shod walking, or the hypothesis that effective mass and joint flexion are similar between footwear conditions.

Among both the Tarahumara and Americans, walking in minimal sandals produced impact peaks that were consistently different from the forces generated by barefoot walking. Specifically, impact peaks generated by walking in minimal sandals had significantly higher magnitudes, slower rates of loading and larger vertical impulses than during barefoot walking. In both populations, the effects of minimal footwear on impact peaks were due, at least partly, to individuals' greater effective mass during the period of impact than in barefoot walking [13]. Individuals also tended to heel strike with slightly more dorsiflexed ankles when in sandals compared to barefoot, which conceivably might have moderately decreased ankle compliance [13]. Together, these results show that, regardless of individuals' habituation to minimal footwear or the exact type of minimal footwear worn, people generally tend to tread more lightly when walking barefoot than even in very minimal sandals. Consistent with this idea, recent studies [40–43] have reported that maximum external force magnitudes across the entire step cycle are also lower when people walk barefoot than shod, including in minimal footwear such as flip-flops, which, similar to our findings, contradicts earlier research suggesting that shoes attenuate ground reaction forces during walking [29,30]. As is evidently the case during running [5], the lower forces generated by walking without shoes are probably initiated by the inevitably greater exteroreception provided by bare feet. Consequently, walking in any kind of footwear should not be expected, or claimed by marketers of ‘barefoot shoes', to mimic barefoot walking.

Although the effects of minimal footwear on walking kinetics and kinematics were generally very similar between Tarahumara and American participants, impact peaks generated by walking in the traditional versus commercial sandals worn by the two populations, respectively, were nevertheless distinct. Specifically, while impact peaks produced by barefoot walking did not significantly differ between groups, Tarahumara walking in traditional sandals generated impact peaks with significantly higher rates of loading and with smaller impulses than Americans wearing commercial sandals. As discussed above, such differences between Tarahumara and American impact peaks during shod walking might be due to variation in the stiffness of the soles of the different sandals worn by the two groups. Mechanical testing revealed that Tarahumara sandal soles are at least 2.4-fold stiffer than those of the Americans' sandals, and controlled laboratory experiments have shown that while stiffer soles have little effect on the magnitude of loading [15,29,30], they increase the rate of collision between the foot and ground, inevitably decreasing the impulse [15]. Nevertheless, because all participants were measured walking only in a single type of minimal footwear, it is possible that differences between impact peaks produced by minimally shod Tarahumara and Americans were partly due to group differences in walking biomechanics rather than just variation in sandal sole stiffness. Individuals were not measured wearing both types of sandals because most Americans are unlikely to buy sandals made of car tyre treads, and the Tarahumara do not have access to sandals made from EVA. Ultimately, our goal was not to test whether both populations would respond identically to the same sandals, but instead to examine if the effects of wearing minimal sandals on impact peaks are generally similar across discrete populations, which appears to be the case. Future work is required to more rigorously study the consequences of variation in sole stiffness for impact forces across minimal footwear designs, both commercial types and those maintained by indigenous cultural traditions.

The extent to which impact peaks generated by walking (and running) might contribute to musculoskeletal disorders has been vigorously debated [24,26–28] and was not tested by this study. Nevertheless, even if certain kinds of impact peaks pose some degree of added risk to musculoskeletal health, from an evolutionary perspective, one would not expect all impact peaks to cause musculoskeletal damage. Bipedal humans and earlier hominins have been experiencing repetitive impact peaks for millions of years, and for the vast majority of that time, these species exclusively walked barefoot [1]. Given that hunter-gatherers walk on average 10–14 km day^−1^ [44], we estimate that our barefoot ancestors during prehistoric times typically endured 4.8–6.7 million impact peaks per year. Even so, there is no indication from the fossil record that these prehistoric ancestors were at any greater risk of musculoskeletal degenerative disorders than less physically active people today [1,6,45], suggesting that human bodies have long been adequately adapted to withstand the low-magnitude, high-rate and low-impulse impact loading characteristic of barefoot walking over a long lifetime [46]. To some degree, such impacts may even have some beneficial effects on musculoskeletal health [47,48]. However, further research is needed to test if the higher-magnitude, lower-rate and higher-impulse impact loading characteristic of shod walking may potentially lead to negative effects.

Although minimal footwear does not replicate the impact forces of barefoot walking, there is evidence that a potential benefit of minimal shoes is to strengthen the foot [20,49–51]. Prospective control studies of American adults have shown that individuals who transition to minimal running shoes develop larger intrinsic foot muscles [49,50] and higher longitudinal arches [49], and a retrospective study of Tarahumara and American adults revealed that the use of minimal footwear throughout life is associated with larger intrinsic foot muscles and dynamically stiffer feet during walking [51]. Stronger intrinsic foot muscles may protect the hard and soft tissue structures of the foot and leg from damage under high loads [52].

This study has at least three notable limitations. First, all participants were males because we were unable to collect sufficient data from Tarahumara females. However, there is no reason to expect impact forces to differ between males and females after correcting for body size, and thus we do not believe that the inclusion of females would have yielded dramatically different results. Second, we measured impact peak forces using a pedography platform with a lower sampling frequency than the force plate sensors typically used to measure ground reaction forces in laboratory-based research, and the video camera we used to measure impact velocity had a maximum recording rate that was lower than the rates used in prior studies [13–15]. For these technical reasons, as well as the fact that participants in this study walked at self-selected speeds rather than a fixed speed, directly comparing our kinetic and kinematic data to laboratory-derived data on human walking requires some caution. Nevertheless, our comparisons between barefoot and minimally shod walking should be unbiased by these issues. Third, an inherent limitation to all studies in which ground reaction forces are measured, either with a pedography platform or force plate, is that the material properties of the substrate colliding with the foot differ from the surface properties that humans typically walk and run on, especially the natural surfaces of rural environments. Future studies should consider how natural surfaces affect impact forces when walking barefoot and in minimal footwear [31].

In summary, our results indicate that minimal footwear alters impact peak forces and limb kinematics from those generated during barefoot walking. Therefore, minimal footwear should not be assumed to expose human bodies to the same biomechanical environment as walking without shoes. These differences hold true regardless of whether a person has worn minimal footwear for their entire life, like the Tarahumara studied here, or if a person has little experience with minimal footwear, like the American participants in this study. The results also raise the possibility that the material of the sole of different types of minimal footwear may be an important influence on how impact forces are modulated, with stiffer materials resulting in higher impact loading rates and lower impulses of loading. This hypothesis, which requires further testing, implies that stiffer soled footwear more closely approximates the impact environment experienced by the foot during barefoot walking. Given that human anatomy evolved to cope with the forces of barefoot walking over millions of years, it may be beneficial to use footwear that least alters these forces, but further research on how impact forces affect musculoskeletal health is necessary to test this hypothesis.

## Supplementary Material

Supporting data

## Data accessibility

All data are available in the online electronic supplementary material.
